# Differences in the epigenetic regulation of MT-3 gene expression between parental and Cd^+2 ^or As^+3 ^transformed human urothelial cells

**DOI:** 10.1186/1475-2867-11-2

**Published:** 2011-02-08

**Authors:** Seema Somji, Scott H Garrett, Conrad Toni, Xu Dong Zhou, Yun Zheng, Amornpan Ajjimaporn, Mary Ann Sens, Donald A Sens

**Affiliations:** 1 Department of Pathology, School of Medicine and Health Sciences, University of North Dakota, Grand Forks, ND, USA; 2 Sanford Health, Fargo, ND, USA

## Abstract

**Background:**

Studies have shown that metallothionein 3 (MT-3) is not expressed in normal urothelium or in the UROtsa cell line, but is expressed in urothelial cancer and in tumors generated from the UROtsa cells that have been transformed by cadmium (Cd^+2^) or arsenite (As^+3^).The present study had two major goals. One, to determine if epigenetic modifications control urothelial MT-3 gene expression and if regulation is altered by malignant transformation by Cd^+2 ^or As^+3^. Two, to determine if MT-3 expression might translate clinically as a biomarker for malignant urothelial cells released into the urine.

**Results:**

The histone deacetylase inhibitor MS-275 induced MT-3 mRNA expression in both parental UROtsa cells and their transformed counterparts. The demethylating agent, 5-Aza-2'-deoxycytidine (5-AZC) had no effect on MT-3 mRNA expression. ChIP analysis showed that metal-responsive transformation factor-1 (MTF-1) binding to metal response elements (MRE) elements of the MT-3 promoter was restricted in parental UROtsa cells, but MTF-1 binding to the MREs was unrestricted in the transformed cell lines. Histone modifications at acetyl H4, trimethyl H3K4, trimethyl H3K27, and trimethyl H3K9 were compared between the parental and transformed cell lines in the presence and absence of MS-275. The pattern of histone modifications suggested that the MT-3 promoter in the Cd^+2 ^and As^+3 ^transformed cells has gained bivalent chromatin structure, having elements of being "transcriptionally repressed" and "transcription ready", when compared to parental cells. An analysis of MT-3 staining in urinary cytologies showed that a subset of both active and non-active patients with urothelial cancer shed positive cells in their urine, but that control patients only rarely shed MT-3 positive cells.

**Conclusion:**

The MT-3 gene is silenced in non-transformed urothelial cells by a mechanism involving histone modification of the MT-3 promoter. In contrast, transformation of the urothelial cells with either Cd^+2 ^or As^+3 ^modified the chromatin of the MT-3 promoter to a bivalent state of promoter readiness. Urinary cytology for MT-3 positive cells would not improve the diagnosis of urothelial cancer, but might have potential as a biomarker for tumor progression.

## Background

This laboratory has proposed the third isoform of the metallothionein gene family as a potential biomarker for the development of human bladder cancer [1,2]. This was first suggested by a retrospective immunohistochemical analysis of MT-3 expression on a modest sample set of archival diagnostic specimens composed of benign and cancerous lesions of the bladder [1]. The cells of the normal bladder were shown to have no immunoreactivity for the MT-3 protein, and no expression of MT-3 mRNA or protein were noted in extracts prepared from samples from surgically removed normal bladder tissue. In contrast, all specimens of urothelial cancer were immunoreactive for the MT-3 protein, and the intensity of staining correlated to tumor grade. This was later expanded to a more robust retrospective study using archival diagnostic tissue [2]. This study showed that only 2 of 63 (3.17%) benign bladder specimens had even weak immunostaining for the MT-3 protein. In contrast, 103 of 107 (96.26%) high grade urothelial cancers and 17 of 17 (100%) specimens of carcinoma *in situ *stained positive for the MT-3 protein. For low grade urothelial cancer, 30 of 48 specimens (62.5%) expressed the MT-3 protein.

The laboratory has used the UROtsa cell line as a model system to elucidate the differences in the expression of the MT-3 gene between normal and malignant urothelium. The UROtsa cell line is derived from a primary culture of human urothelial cells that was immortalized using the SV40 large T-antigen [3,4]. The UROtsa cells retain a normal cytogenetic profile, grow as a contact inhibited monolayer, and are not tumorigenic as judged by the inability to form colonies in soft agar and tumors in nude mice. This laboratory showed that UROtsa cells grown in a serum-free growth medium displayed features consistent with the intermediate layer of the urothelium [5]. Identical to that of normal *in situ *urothelium, the UROtsa cell line was shown to have no basal expression of MT-3 mRNA or protein. The laboratory has also directly malignantly transformed the UROtsa cell line by exposure to Cd^+2 ^or As^+3 ^and shown that the tumor transplants produced by the transformed cells had histologic features consistent with human urothelial cancer [6]. An interesting finding in subsequent studies was that MT-3 mRNA and protein was not expressed in the Cd^+2 ^and As^+3 ^transformed cell lines, but was expressed in the tumor transplants generated by these cell lines in immunocompromised mice [2]. That this was not an anomaly of the UROtsa cell line was suggested by identical findings between cell lines and tumor transplants for the MCF-7, T-47 D, Hs 578T, MDA-MB-231 breast cancer cell lines and the PC-3 prostate cancer cell lines [2]. The first goal of the present study was to determine if epigenetic modifications were responsible for gene silencing of MT-3 in the parental UROtsa cell line. The second goal of the study was to determine if the accessibility of the MRE of the MT-3 promoter to the MTF-1 transcription factor was different between the parental UROtsa cell line and the UROtsa cell lines malignantly transformed by either Cd^+2 ^or As^+3^. The third goal was to determine if histone modifications were different between the parental UROtsa cell line and the transformed cell lines. The last goal was to perform a preliminary analysis to determine if MT-3 expression might translate clinically as a possible biomarker for malignant urothelial cells released into the urine by patients with urothelial cancer.

## Results

### MT-3 mRNA expression following treatment of parental UROtsa cells and their Cd^+2 ^and As^+3 ^transformed counterparts with inhibitors of DNA methylation and acetylation

**Figure 1:**
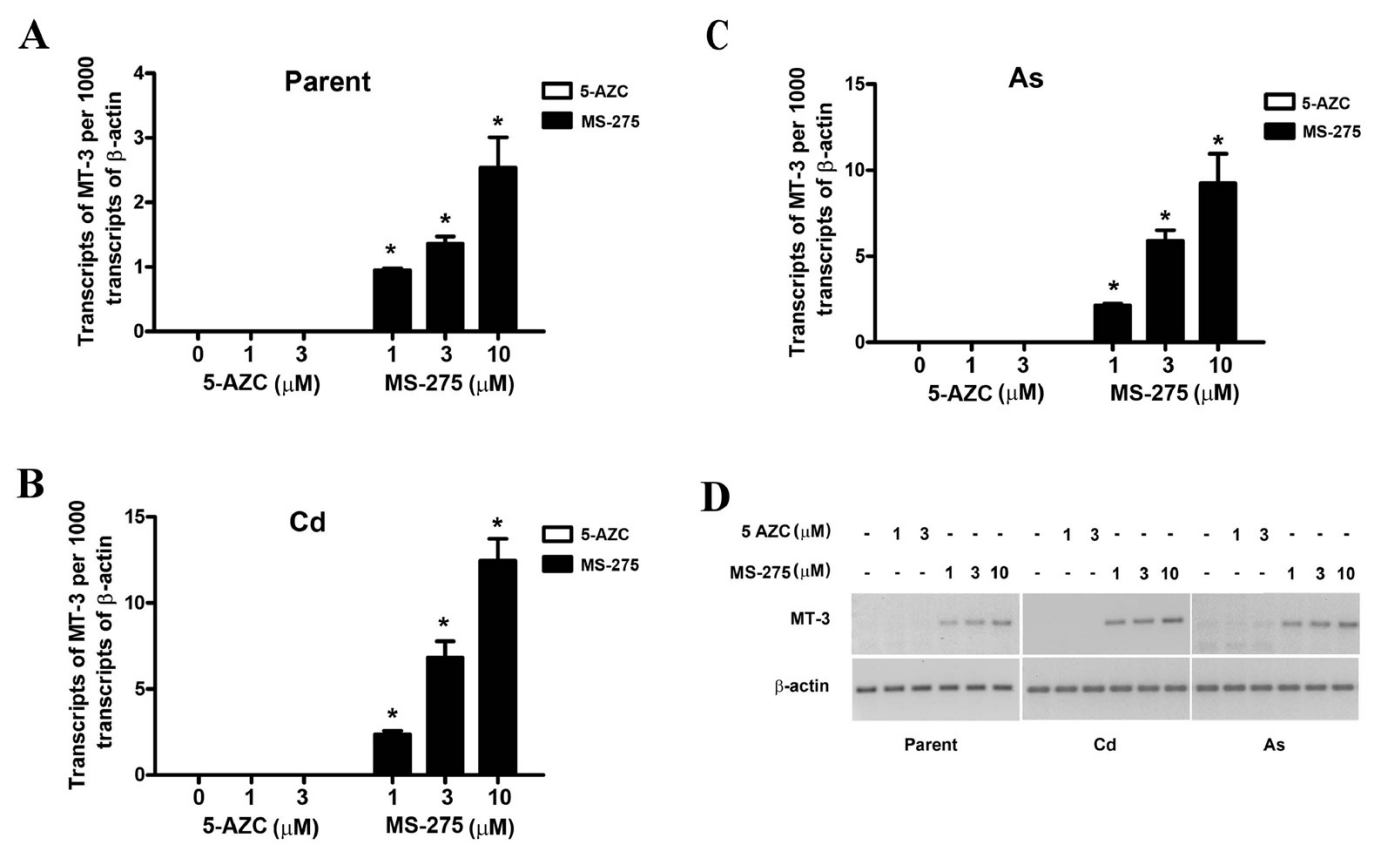
**Expression of MT-3 mRNA in UROtsa parent, Cd^+2 ^and As^+3 ^transformed cell lines**. UROtsa parent and the transformed cell lines were seeded at a 1:10 ratio in the presence of MS-275 till they reached confluency and the expression of MT-3 was determined by RT-PCR analysis. A. Real time PCR analysis of MT-3 in UROtsa parent cells. B. Real time PCR analysis of MT-3 in Cd^+2 ^transformed UROtsa cells. C. Real time PCR analysis of MT-3 in As^+3 ^transformed UROtsa cells. The expression of MT-3 was normalized to that of β-actin. The determinations were performed in triplicates and the results shown are the mean ± SE. * Statistically significant compared to untreated control cells. D. Ethidium bromide stained gel showing the expression of MT-3 in the UROtsa cell lines by semiquantitativePCR.

The parental and transformed UROtsa cells were treated with the histone deacetylase inhibitor, MS-275, and the methylation inhibitor 5-AZC, to determine the possible role of histone modifications and DNA methylation on MT-3 mRNA expression. In the initial determinations, subconfluent cells were treated with either MS-275 or 5-AZC and allowed to proliferate to confluency, at which time they were harvested for the determination of MT-3 mRNA expression. This analysis demonstrated that parental UROtsa cells treated with MS-275 expressed increased levels of MT-3 mRNA compared to control cells (Figure 1A, D). There was a dose response relationship with a peak in MT-3 expression at a 10 μM concentration of MS-275, the highest concentration which showed no toxicity and allowed the cells to attain confluency. MS-275 was dissolved in DMSO and it was shown that DMSO had no effect on MT-3 mRNA expression in parental UROtsa cells (data not shown). An identical treatment of the Cd^+2 ^and As^+3 ^transformed UROtsa cells with MS-275 also demonstrated increased MT-3 mRNA levels and a similar dose response relationship to that of the parental cells (Figure 1B, C, D). The increase in MT-3 mRNA expression due to MS-275 treatment was several fold greater in the Cd^+2 ^and As^+3 ^transformed UROtsa cells compared to that of the parental cells. It was also shown that DMSO had no effect on MT-3 expression in the transformed cell lines and that MS-275 had no toxicity similar to that of the parental cells. In contrast, a similar treatment of the parental UROtsa cells or their transformed counterparts with the demethylating agent, 5-AZC, had no effect on the expression of MT-3 mRNA over that of untreated cells (Figure 1A-D). Concentrations of 5-AZC were tested up to and including those that inhibited cell proliferation and no increase in MT-3 expression was found at any concentration.

**Figure 2:**
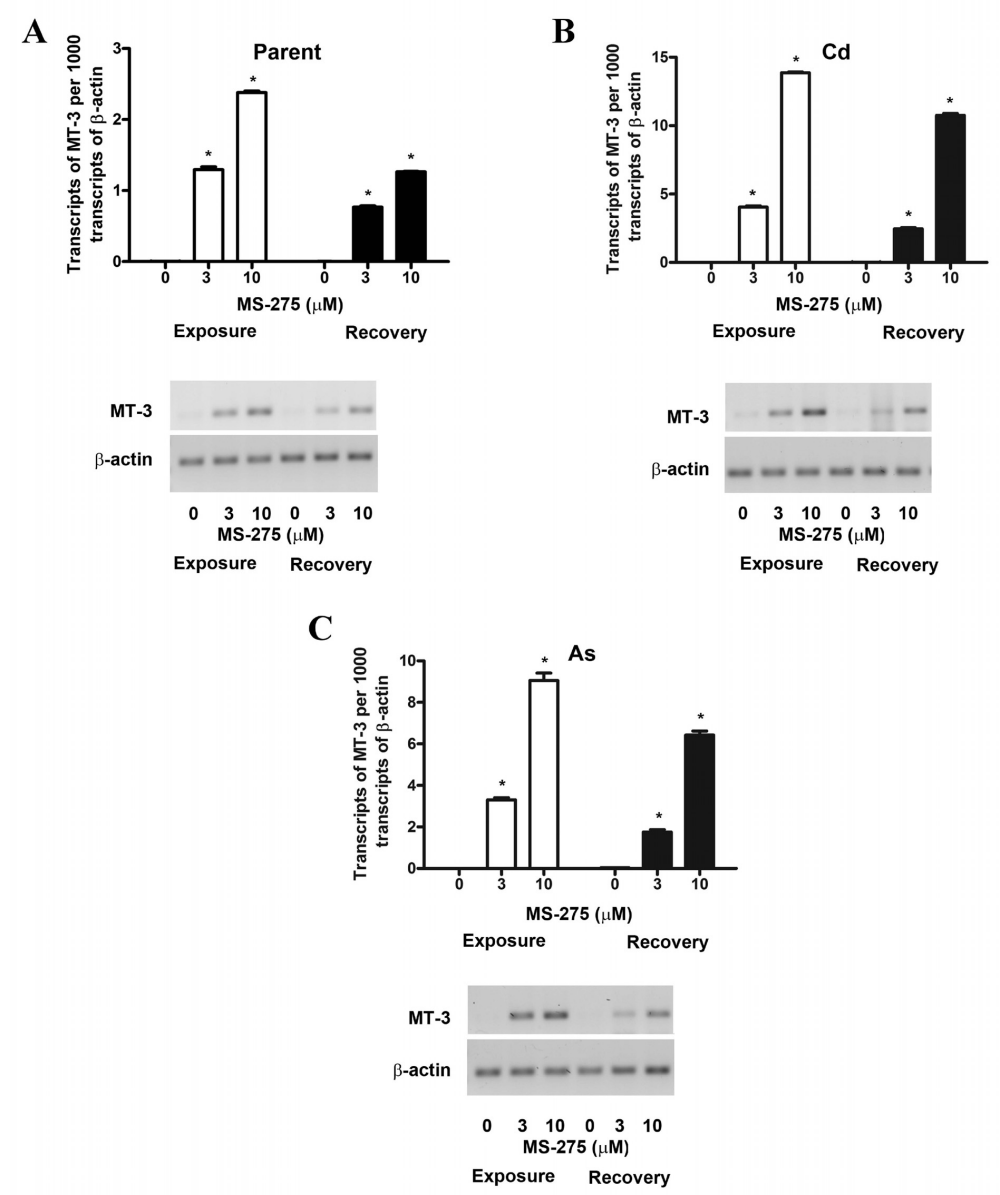
**Expression of MT-3 in parent, Cd^+2 ^and As^+3 ^transformed cells after removal of MS-275**. Cells were cultured with 10 μM MS-275 until they reached confluency after which the drug was removed and the cells were allowed to recover for 24 h following which RNA was extracted from the recovered cells. A. RT-PCR analysis of MT-3 expression in UROtsa parent cells. B. RT-PCR analysis of MT-3 expression in Cd^+2 ^transformed UROtsa cells. C. RT-PCR analysis of MT-3 expression in As^+3 ^transformed UROtsa cells. Graphs represent real time RT-PCR data whereas ethidium bromide stained gels show the semiquantitative PCR analysis data. The expression of MT-3 was normalized to that of β-actin. The determinations were performed in triplicates and the results shown are the mean ± SE.*Statistically significant compared to untreated control cells.

A second determination was performed to determine if initial treatment of the parental and transformed UROtsa cells with MS-275 would allow MT-3 mRNA expression to continue after removal of the drug. In this experiment, the cells were treated with MS-275 as above, but the drug was removed when the cells attained confluency and MT-3 expression determined 24 h after drug removal. This determination showed that MT-3 expression was still elevated following drug removal for the parental UROtsa cells and their transformed counterparts, albeit, at modestly reduced levels of expression for all three cell lines (Figure 2A-C). There was no difference in the degree of reduction of MT-3 expression between the cells lines nor between the treatment and recovery periods.

### Differences in zinc induction of MT-3 mRNA expression between normal and transformed UROtsa cells following inhibition of histone deacetylase activity

**Figure 3:**
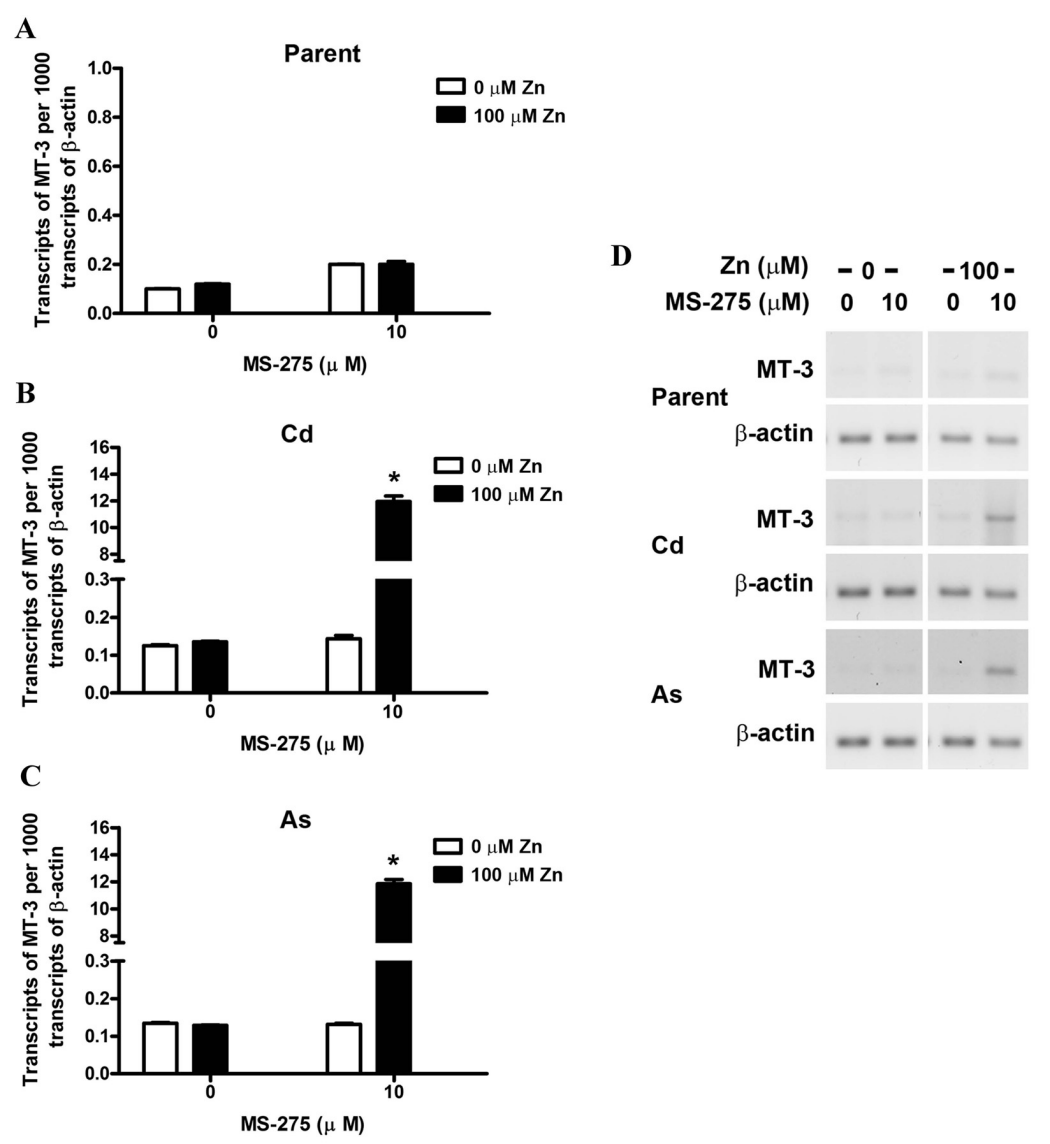
**Effect of Zn^+2 ^on MT-3 levels in the parent and transformed UROtsa cell lines**. UROtsa parent and the transformed cell lines were seeded at a 1:10 ratio in the presence of MS-275 until they reached confluency, following which the cells were allowed to recover for 24 h without the drug. The cells were then exposed to 100 μM zinc for 24 h and RNA was extracted. A. Real time RT-PCR analysis of MT-3 expression in UROtsa parent cells. B. Real time RT-PCR analysis of MT-3 expression in Cd^+2 ^transformed UROtsa cells. C. Real time RT-PCR analysis of MT-3 expression in As^+3 ^transformed UROtsa cells. The expression of MT-3 was normalized to that of β-actin. The determinations were performed in triplicates and the results shown are the mean ± SE.*Statistically significant compared to untreated control cells. D. Ethidium bromide stained gel showing the expression of MT-3 in the UROtsa cell lines using semiquantitativePCR.

As described above, the parental and transformed UROtsa cells were allowed to proliferate to confluency in the presence of MS-275 and then allowed to recover for 24 h in the absence of the drug. After the recovery period, the cells were then exposed to 100 μM zinc for 24 h and prepared for the analysis of MT-3 mRNA expression. The parental UROtsa cells previously exposed to MS-275 showed no increase in MT-3 mRNA expression when treated with 100 μM Zn^+2 ^for 24 h (Figure 3A, D). In contrast, MT-3 expression was induced over a 100 fold when the Cd^+2 ^and As^+3 ^transformed cell lines that had been previously treated with MS-275 were exposed to 100 μM Zn^+2 ^(Figure 3B, C, D).

### **Histone modifications associated with the MT**-**3 promoter in the UROtsa parent and transformed cell lines**

**Figure 4:**
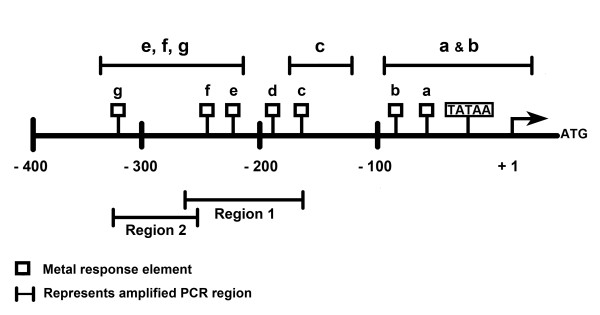
**Schematic illustration of the MT-3 promoter**. The distributions of the MREs and the designated amplified regions are indicated.

**Figure 5:**
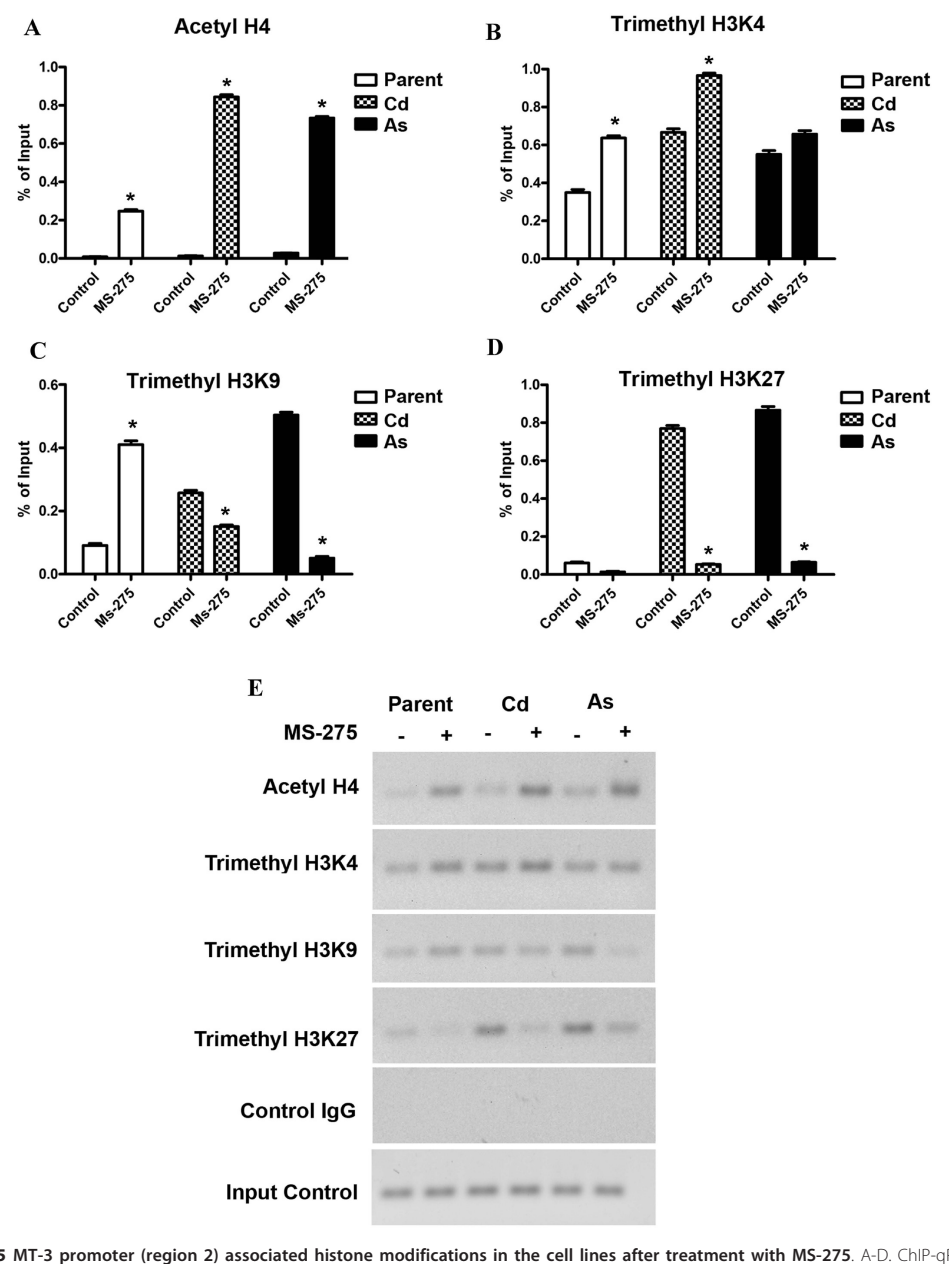
**MT-3 promoter (region 2) associated histone modifications in the cell lines after treatment with MS-275**. A-D. ChIP-qPCR of acetyl H4, trimethy H3K4, trimethyl H3K9 and trimethyl H3K27 at the MT-3 promoter using primers for region 2. The amplification value of the immunoprecipitated DNA was normalized to percentage of input (non-precipitated DNA). The determinations were performed in triplicates and the results shown are the mean ± SE.*Statistically significant compared to untreated control cells. E. Ethidium bromide stained gel showing the amplification of the histone modifications using semiquantitative PCR.

Two regions of the MT-3 promoter were analyzed for histone modifications before and after treatment of the respective cell lines with MS-275. These were chosen to be regions containing sequences of the known metal response elements. The first region chosen spans the largest cluster of MREs (MREc, d, e, and MREf) and is designated as region 1. The second region is immediately upstream from region 1, extends up to and includes MREg and is designated region 2 (Figure 4). The level of acetyl H4, trimethyl H3K4, trimethyl H3K9 and trimethyl H3K27 modifications were determined for each of the two regions of the MT-3 promoter using ChIP-qPCR. In the distal region 2, it was shown that the modification of acetyl H4 was increased in the parental UROtsa cells and both transformed cell lines following treatment with MS-275 (Figure 5A-E). For all three cell lines, there was only a marginal modification for acetyl H4 in cells not treated with MS-275. In addition, the relative increase in acetyl H4 modification following MS-275 treatment was greater in the Cd^+2 ^and As^+3 ^transformed cell line compared to parental cells. There was modification of trimethyl H3K4 in both the normal and transformed UROtsa cell lines under basal conditions and the level of modification increased for the parental UROtsa cells and the Cd^+2 ^transformed cell line following treatment with MS-275 (Figure 5B, E). There was no increase in the level of modification of H3K4 following MS-275 treatment of the As^+3 ^transformed UROtsa cells. Modification of trimethyl H3K9 was present in both the parental and transformed UROtsa cells under basal conditions (Figure 5C, E). The basal level of H3K9 modification was increased for both transformed cell lines when compared to parental cells and also when the As^+3 ^transformed cell line was compared to the Cd^+2 ^transformed cell line. There was a differential response in the level of H3K9 modification when the cells were treated with MS-275. The parental UROtsa cells showed an increase in the modification of H3K9 following MS-275 treatment; whereas, both transformed cell lines showed a decrease in the level of H3K9 modification. The relative magnitude of these differences was large for the parental and As^+3 ^transformed cell lines. There was a large difference in the level of modification of H3K27 between the parental and the transformed cell lines, with the parent having a very low level and the transformed lines highly elevated in their modification of H3K27 (Figure 5D, E). Treatment of both the Cd^+2 ^and As^+3 ^transformed cell lines with MS-275 resulted in a large decrease in the level of H3K27 modification; returning to a level similar to that found in parental cells.

**Figure 6:**
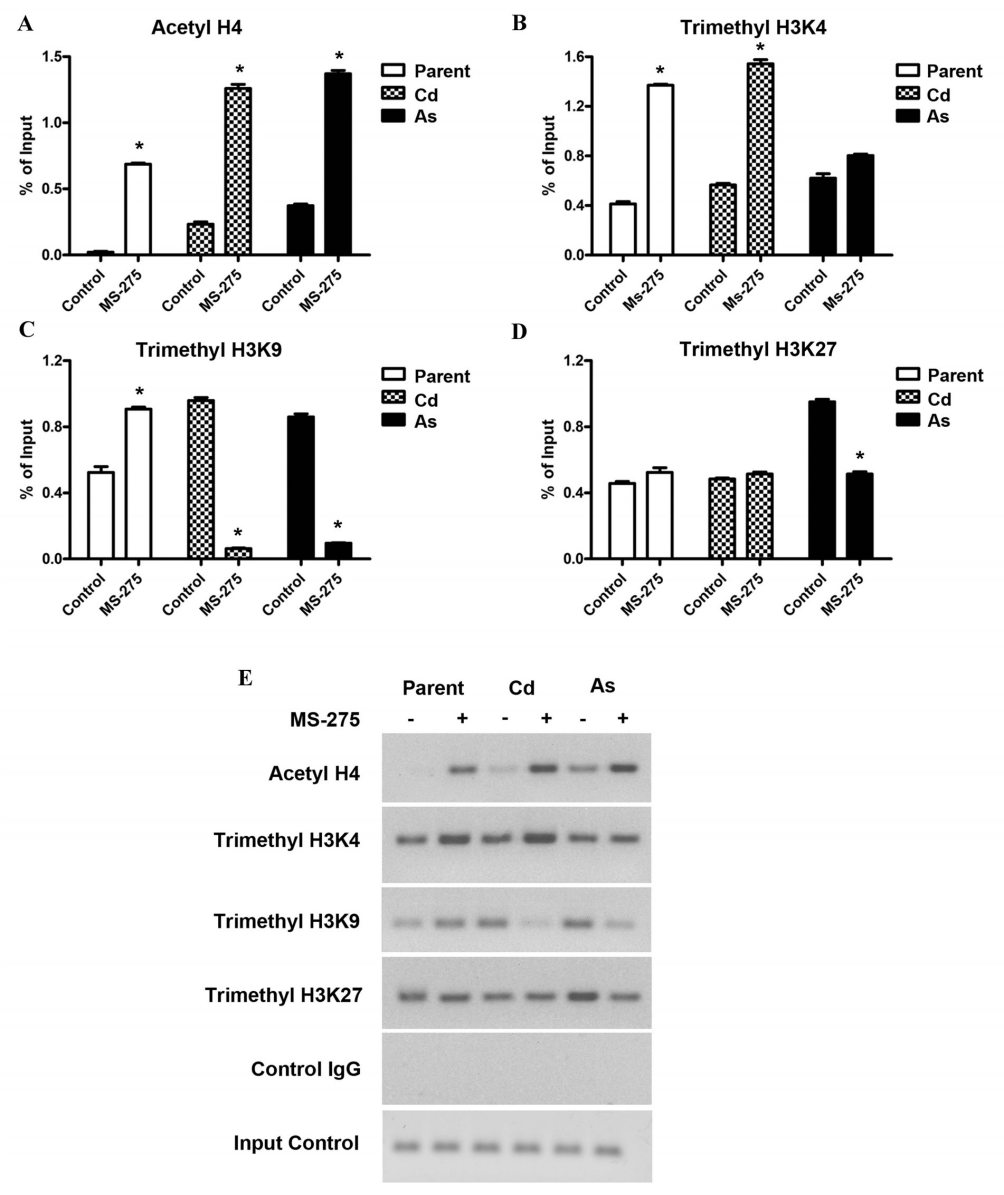
**MT-3 promoter (region 1) associated histone modifications in the cell lines after treatment with MS-275**. A-D. ChIP-qPCR of acetyl H4, trimethy H3K4, trimethyl H3K9 and trimethyl H3K27 at the MT-3 promoter using primers for region 1. The amplification value of the immunoprecipitated DNA was normalized to percentage of input (non-precipitated DNA). The determinations were performed in triplicates and the results shown are the mean ± SE.*Statistically significant compared to untreated control cells. E. Ethidium bromide stained gel showing the amplification of the histone modifications using semiquantitative PCR.

In themore proximal, down-stream promoter region 1, the modification pattern of acetyl H4 was similar to that of region 2, with the exception that the basal level of modification was increased in the Cd^+2 ^and As^+3 ^transformed cell lines (Figure 6A, E). The modification pattern of trimethyl H3K4 was also similar between the two promoter regions with only subtle alterations in the level of modification (Figure 6B, E). The pattern of trimethyl H3K9 modification was also similar between the two promoter regions, with the exception that the basal modification of trimethyl H3K9 was increased in the Cd^+2 ^transformed cell line (Figure 6C, E). There were significant differences in the modification of trimethyl H3K27 between the two promoter regions from the cell lines. There was modification of trimethyl H3K27 in the parental UROtsa cells in the absence of MS-275 treatment and the level of modification did not change with MS-275 treatment (Figure 6D, E). The extent of modification of trimethyl H3K27 in the Cd^+2 ^transformed cells was identical to the parental cells. The modification of trimethyl H3K27 was reduced by MS-275 treatment in the As^+3 ^transformed cells, but to a lesser degree than noted for the proximal promoter.

### **Histone modification and competency of MTF**-**1 binding to the MREs of the MT-3 promoter in normal and transformed UROtsa cells**

**Figure 7:**
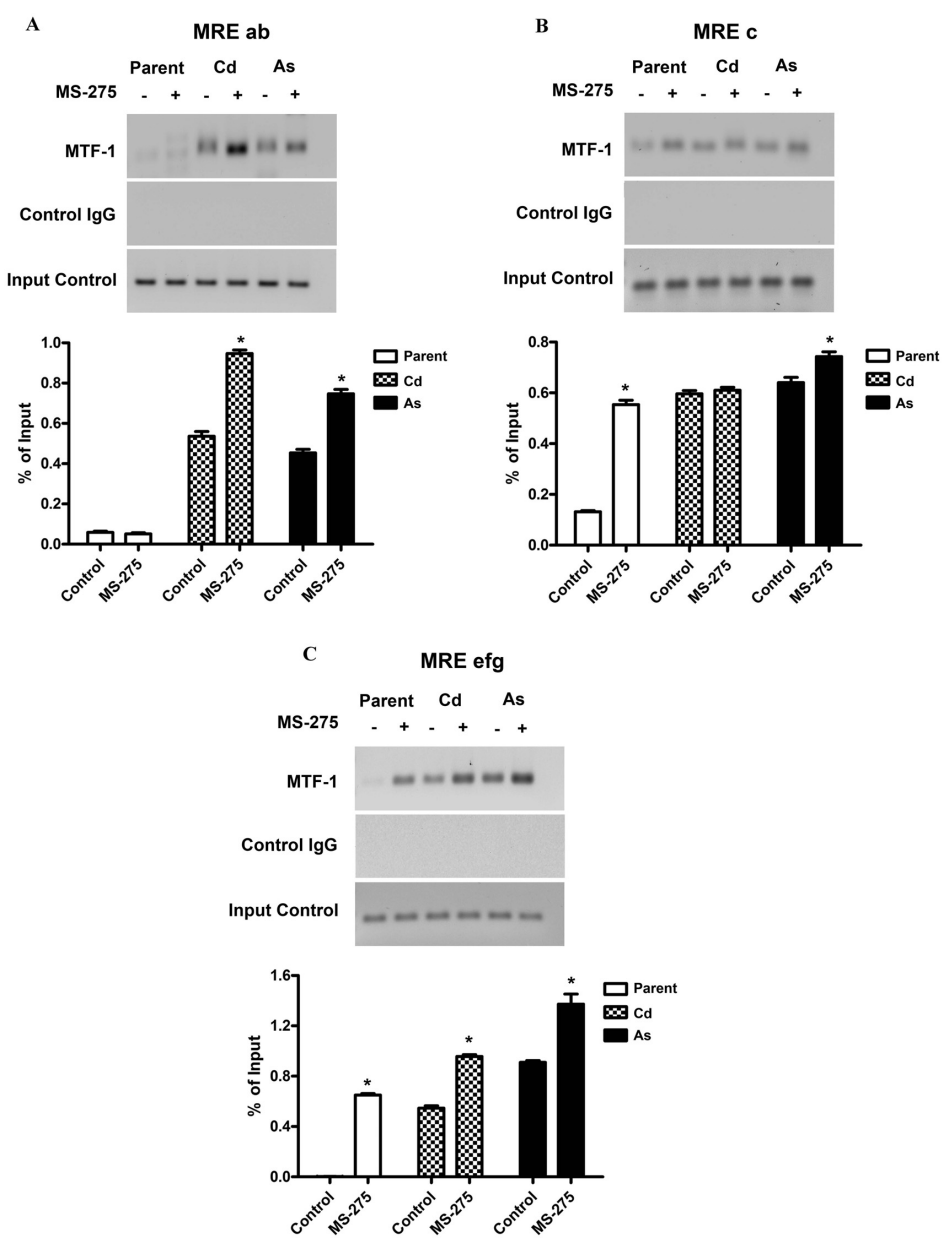
**Recruitment of MTF-1 to the MREs of the MT-3 promoter after treatment with MS-275**. UROtsa parent and transformed cells were seeded at a 1:10 ratio and were grown in the presence of 10 μM MS-275 until they reached confluency. They were then exposed to 100 μM Zn^+2 ^for 4 h and the chromatin was immunoprecipitated with the MTF-1 antibody. PCR was performed with primers specific to each region as specified in Table 2 and Figure 4 to assess the level of precipitated sequences. A. ChIP-q PCR analysis of MTF-1 recruitment to MRE a and MREb. B. ChIP-q PCR analysis of MTF-1 recruitment to MRE-c. C. ChIP-q PCR analysis of MTF-1 recruitment to MREe, MREf and MREg. The amplification value of the immunoprecipitated DNA was normalized to percentage of input (non-precipitated DNA). The determinations were performed in triplicates and the results shown are the mean ± SE.*Statistically significant compared to untreated control cells. Graphs represent real time PCR data whereas ethidium bromide stained gels represent semiquantitative PCR data.

The ability of MTF-1 to bind the MRE elements of the MT-3 promoter was determined in the parental UROtsa cell line and the Cd^+2 ^and As^+3 ^transformed cell lines before and after treatment with MS-275. Primers were designed to break the MREs down to as many individual measureable units as possible. Only specific primers for three regions were possible as designated in Figure 1. The results of this analysis showed that there was little or no binding of MTF-1 to the MREa or MREb sequences in the MT-3 promoter of the parental UROtsa cells with or without treatment with MS-275 (Figure 7A). In contrast, the MREa, b elements of MT-3 promoter in the Cd^+2 ^and As^+3 ^transformed cell lines were able to bind MTF-1 under basal conditions and with increased efficiency following treatment with MS-275 (Figure 7A). A similar analysis of the MREc element in the MT-3 promoter showed a low amount of MTF-1 binding to parental UROtsa cells not treated with MS-275 and a significant increase in binding following treatment with MS-275 (Figure 7B). The Cd^+2 ^and As^+3 ^transformed cell lines showed appreciable MTF-1 binding to the MREc element of the MT-3 promoter in the absence of MS-275 when compared to the parental UROtsa cells. Treatment with MS-275 had no further effect on MTF-1 binding to the MREc element of the MT-3 promoter for the Cd^+2 ^transformed cells and only a small increase for the As^+3 ^transformed cells (Figure 7B). There was no binding of the MTF-1 to the MREe, f, g elements of the MT-3 promoter for parental UROtsa cells unexposed to MS-275 (Figure 7C). In contrast, there was binding when the parental UROtsa cells were treated with MS-275. There was binding of MTF-1 to the MREe, f, g elements of the MT-3 promoter in both Cd^+2 ^and As^+3 ^transformed cell lines under control conditions and a further increase in binding when the cell lines were treated with MS-275 (Figure 7C).

### Presence of MT-3 positive cells in urinary cytologies of patients with bladder cancer

Urine samples were collected and urinary cytologies prepared over a 5 year period on patients attending the regularly scheduled urology clinic. A total of 276 urine specimens were collected in the study with males comprising 67% of the total samples and the average patient age was 70.4 years with a distribution of 20 to 90 years of age. The control group was defined as individuals attending the urology clinic for any reason other than a suspicion of bladder cancer. A total of 117 control samples were collected and of these 60 had cells that could be evaluated by urinary cytology and 57 control samples provided no cells. Only 3 specimens from the control group were found to contain cells that were immunostained for the MT-3 protein (Table 1). Urinary cytologies for 127 patients with a previous history of urothelial cancer, but with no evidence of active disease, were examined and 45 were found to have MT-3 stained cells in their urine. No evidence of active disease was defined by a negative examination of the bladder using cystoscopy (Table 1). There were 32 patients that were confirmed to have active disease by cystoscopy and of these, 19 were found to have MT-3 positive cells by urinary cytology (Table 1). There were significant differences between the control and recurrence group of patients, the control versus non recurrence group and the recurrence versus no recurrence group as determined by the Pearson Chi-square test.

**Table 1:** MT-3 expression in urinary cytology specimens

Group	n	MT-3 positive	(%) Percentage
Control***^a^***,***^b^***	117	3	2.6

Inactive Disease***^a^***,***^c^***	127	45	35.4

Active Disease***^b^***,***^c^***	32	19	59.4

***^a^***Control vs. Active; Pearson Chi-square 33.91 p = 0.000

***^b^***Control vs. Inactive; Pearson Chi-square 19.78 p = 0.000

***^c^***Active vs. Inactive; Pearson Chi-square6.09p = 0.014

**Figure 8:**
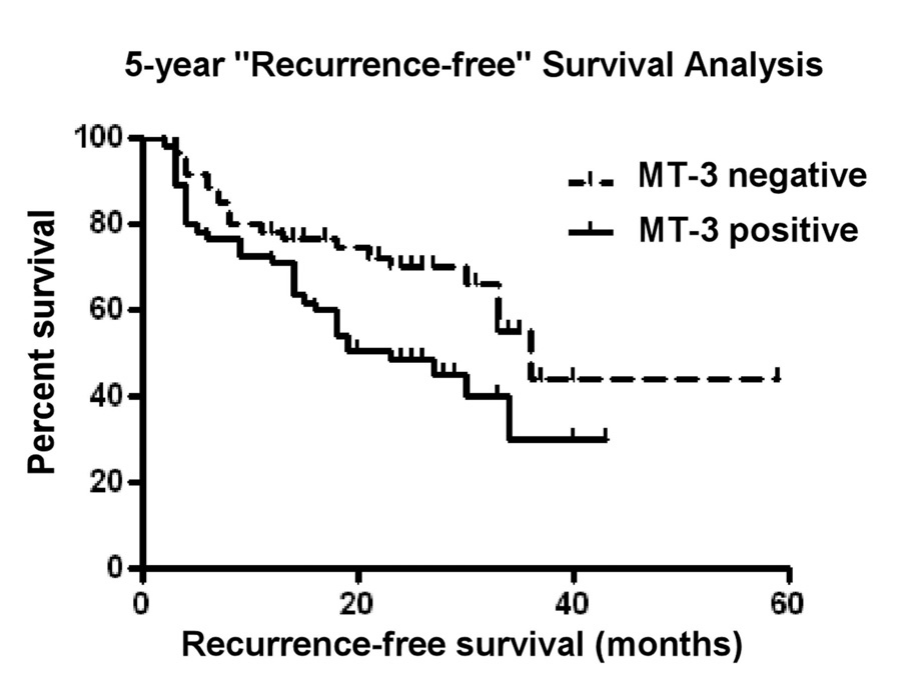
**Kaplan Meier plot demonstrating prolonged survival of MT-3 negative bladder cancer**. The survival distributions between MT-3 positive (solid line) and MT-3 negative (dotted line) bladder cancer patients. The data shows a statistically significant difference (Log Rank 5.067, *p *= 0.024; Tarone-Ware 5.071; *p *= 0.024).

There were 90 patients in the study (115 urine cytologies) that had either multiple urine collections on return visits to the clinic, or who had previously provided a urine specimen and later returned to the clinic for follow-up but without providing a urine specimen for the study. These were able to be followed for recurrence of urothelial cancer from 2 months up to 59 months. This allowed an analysis of 18 recurrences and 29 non-recurrences in those yielding cytologies with MT-3 positive cells and 7 recurrences and 24 non-recurrences in those yielding cytologies with no MT-3 positive cells. A comparison of the time to recurrence between these two groups revealed a significant statistical difference (log rank 5.067, p = 0.024 and Tarone-Ware 5.071, p = 0.024) between those with urinary cytologies with MT-3 staining cells and those with no MT-3 staining cells (Figure 8).

## Discussion

The initial goal of this study was to determine if epigenetic modification was responsible for the silencing of the MT-3 gene in the parental UROtsa cell line. Treatment of the parental UROtsa cells with 5-AZC, a commonly used agent to determine DNA methylation status, was shown to have no effect on MT-3 mRNA expression. This provides evidence that the MT-3 gene was not silenced by a mechanism involving DNA methylation in the parental UROtsa cells. The treatment of the cells with MS-275, a histone deacetylase inhibitor, was shown to result in the expression of MT-3 mRNA by the parental UROtsa cell line. MS-275 has been shown to preferentially inhibit HDAC 1 compared to HDAC 3 and has little or no effect on HDAC 6 and 8 [7-9]. This finding provides strong evidence that MT-3 expression is silenced in the parental UROtsa cell line through a mechanism involving histone modification. The MT-3 gene is also silent in cell lines derived from the UROtsa parent that have been malignantly transformed by either Cd^+2 ^or As^+3 ^[2,6]. A pattern of MT-3 mRNA expression similar to that for the parental UROtsa cells was found following treatment of the Cd^+2 ^and As^+3 ^transformed cell lines with 5-AZC and MS-275. The only exception being that the expression of MT-3 mRNA was several fold higher following MS-275 treatment in the Cd^+2 ^and As^+3 ^transformed cell lines compared to the parental UROtsa cells. These findings suggest that MT-3 gene expression is silenced in both the parental UROtsa cells and the Cd^+2 ^and As^+3 ^transformed counterparts through a mechanism involving histone modification.

The second goal of the study was to determine if the accessibility of the MREs of the MT-3 promoter to a transcription factor were different between the parental UROtsa cell line and the UROtsa cell lines malignantly transformed by either Cd^+2 ^or As^+3^. The initial indication that the integrity of the MT-3 promoter may be different between the parent and transformed UROtsa cells, was that MT-3 mRNA expression could be further induced by Zn^+2 ^in the transformed cell lines following treatment with MS-275, but was not induced by an identical treatment in the parental UROtsa cell line. This observation was extended by an analysis of the accessibility of the MREs within the MT-3 promoter to binding of MTF-1. MTF-1 is a constitutively expressed transcription factor that is activated by diverse stress stimuli, the most notable being metal load [10]. Upon stimulation MTF-1 translocates to the nucleus where it binds to the enhancers/promoters of target genes that harbor one or multiple copies of the specific recognition sequence, called MREs. The best characterized of these target genes are the metallothioneins [11]. The analysis was performed in the presence of 100 μM Zn^+2 ^because Zn^+2 ^is necessary for the activation of MTF-1 and 100 μM is the concentration commonly utilized to determine MTF-1 activation. ChIP analysis showed that there was no binding of MTF-1 to MREa and MREb of the MT-3 promoter in the parental UROtsa cell line before or after treatment with MS-275. In contrast, there was MTF-1 binding to MREa and MREb of the MT-3 promoter in the Cd^+2 ^and As^+3 ^transformed cell lines under basal conditions, with a further increase in binding following treatment with MS-275. A similar analysis of MTF-1 binding to MREc in the MT-3 promoter showed the parental cells to have limited binding under basal conditions and an increased interaction following treatment with MS-275. In contrast, the Cd^+2 ^and As^+3 ^transformed cell lines were shown to have increased binding of MTF-1 to MREc of the MT-3 promoter under both basal conditions with no increase in interaction following treatment with MS-275. An identical analysis of MREe, f and g of the MT-3 promoter with MTF-1 showed no interaction in the parental UROtsa cell under basal conditions and an increase in binding following treatment with MS-275. In contrast, MREe, f, g of the MT-3 promoter were able to bind MTF-1 under basal conditions, which was increased following treatment with MS-275. These studies show that there is a fundamental difference in the accessibility of MREs to MTF-1 binding within the MT-3 promoter between the parental UROtsa cells and the Cd^+2 ^and As^+3 ^transformed cell lines. Under basal conditions, the MREs of the MT-3 promoter are not accessible to MTF-1 binding in the parental UROtsa cells. In contrast, the MREs of the MT-3 promoter are accessible for MTF-1 binding under basal conditions in the Cd^+2 ^and As^+3 ^transformed cell lines.

Several common histone modifications, acetyl H4, trimethyl H3K4, trimethyl H3K27, and trimethyl H3K9, associated with gene activation were analyzed in two regions of the MT-3 promoter for the parental UROtsa cells and the Cd^+2 ^and As^+3 ^transformed cell lines. The level of histone H4 acetylation was always increased in both the parental and transformed cell lines in the presence of MT-275. In addition, it was also found to be increased in the more proximal region of the Cd^+2 ^and As^+3 ^transformed cell lines not treated with MS-275 in comparison to the parent cell line. The increase in H4 acetylation correlated with the increase in MT-3 expression and it is known that H4 acetylation is associated with transcriptional activation [12-14]. The antibody used for H4 acetylation does not distinguish among the four potentially acetylated lysines 5, 8, 12, and 16, but all are thought to be involved in transcriptional activation. Similarly, the above noted increases in MT-3 expression in the parental and transformed cell lines also was associated with methylation of H3K4, which is a modification also known to occur in promoters of actively transcribing genes [12-14]. Together, these findings give an indication that the MT-3 promoter in the transformed cells has histone modifications that are positive for transcription of the MT-3 gene. In contrast to the above the findings which support a "transcription ready" state, are the findings of increased histone H3K9 and H3K27 methylation, which are both associated with a transcriptionally repressed state [12-14]. Taken together, these findings can be interpreted to suggest that the MT-3 promoter in the Cd^+2 ^and As^+3 ^transformed cells has gained bivalent chromatin structure, that is having elements of being "transcriptionally repressed" and "transcription ready", when compared to parental UROtsa cells [15].

It has been shown previously that the Cd^+2 ^and As^+3 ^transformed cell lines have no expression of MT-3 mRNA under cell culture conditions, but gain MT-3 expression when transplanted as tumors in immune compromised mice [2]. Based on the above histone modifications in the cell lines, this finding would suggest that transplantation of the Cd^+2 ^and As^+3 ^transformed cell lines into an *in vivo *environment further alters the chromatin structure of the MT-3 promoter to a state capable of active transcription of the MT-3 gene. This would suggest that the *in vivo *environment is providing a factor/s that is capable of advancing bivalent chromatin to a fully active state. There is no literature base that allows one to speculate what this factor might be or if it would be expected to be soluble or an insoluble component of the cell matrix.

The last goal of this study was to perform a preliminary analysis to determine if MT-3 expression might translate clinically as a possible biomarker for malignant urothelial cells released into the urine by patients with urothelial cancer. This was tested by the collection of urothelial cells from the urine of patients attending their regularly scheduled appointment in the urology clinic. There was no clinical information available regarding the possible exposure of the patients to metals. Urinary cytologies were prepared using standard clinical laboratory methods and the cells subsequently immunostained for MT-3 positive cells using an MT-3 antibody. The hypothesis was that patients with urothelial cancer would shed MT-3 positive cells into their urine and that the shedding of MT-3 positive cells might identify patients with urothelial cancer and also those whose disease had relapsed to an active state. The present diagnosis of urothelial cancer relies on the visual examination of the bladder using a cystoscope. The results of the present study did not support this initial hypothesis for either newly diagnosed patients or for those being assessed for recurrence of urothelial cancer. Urinary cytology documented MT-3 positive cells in only a subset of patients confirmed to have bladder cancer by cystoscopy and also found many instances of MT-3 positive cells in patients having been diagnosed with urothelial cancer and having no evidence of recurrence upon cytoscopic examination. Despite not advancing the initial hypothesis, there were some potentially important findings in the study. First, it was shown that patients without a diagnosis of urothelial cancer rarely had MT-3 positive cells in their urine. The low rate in the control population is significant since these samples were collected in the urology clinic and there are no or few disease-free patients in such a specialized clinic. This indicates a very low rate of MT-3 expression in individuals without urothelial cancer. Second, the results also showed that a subset of urothelial cancer patients did shed MT-3 positive cells into their urine and those with more progressive urothelial cancer were more prone to shed MT-3 positive cells. This may indicate that MT-3 staining in cytologies from newly diagnosed and recurrent urothelial cancer patients may have promise as a prognostic marker for disease progression. There are two rationales in support of this concept. The first is that urinary cytology depends on the loss of strong cell-to-cell contact between adjacent cells, allowing cells to shed into the urine. As such, MT-3 positive cells in the urine may define urothelial cancers where there has been an extensive loss in cell-to-cell contact and interaction with the surrounding tissue environment. These would be expected to define more aggressive cancers prone to invasion of the bladder wall. A second related rationale involves a field effect of "normal" tissue adjacent to the urothelial cancer that may have expression of MT-3. This would explain the presence of MT-3 positive cells in the urine from individuals negative for a recurrence of bladder cancer when examined by cytoscopy. The field effect would contain pre-malignant cells that are positive for MT-3. A long term clinical follow-up of current patients and further analysis of archival tissue will be necessary to advance these possibilities.

## Conclusions

This study shows that the MT-3 gene is silenced in non-transformed urothelial cells by a mechanism involving histone modification of the MT-3 promoter. In contrast, transformation of the urothelial cells with either Cd^+2 ^or As^+3 ^modified the chromatin of the MT-3 promoter to a bivalent state of promoter readiness. Urinary cytology demonstrated the presence of MT-3 positive cells in the urine of some bladder cancers but did not correlate with active disease status. It was rare to find MT-3 positive cells in the urine from control subjects.

## Methods

### Cell culture

Stock cultures of the parent UROtsa cell line and the transformed Cd^+2 ^and As^+3^cell lines were maintained in 75 cm^2 ^tissue culture flasks using Dulbecco's modified Eagles' medium (DMEM) containing 5% v/v fetal calf serum in a 37°C, 5% CO_2_: 95% air atmosphere [2]. Confluent flasks were sub-cultured at a 1:4 ratio using trypsin-EDTA (0.05%, 0.02%) and the cells were fed fresh growth medium every 3 days.

### Treatment of UROtsa cells with 5-Aza-2'-deoxycytidine and histone deacetylase inhibitor MS-275

Parent and transformed UROtsa cells (10^6^)were seeded at a 1:10 ratio and the next day they were treated with 1 or 3 μM 5-AZC (Sigma-Aldrich, St. Louis, MO) or 1, 3 or 10 μM MS-275 (ALEXIS Biochemicals, Lausen, Switzerland). The cells were allowed to grow to confluency (48 h) and then harvested for RNA isolation. For the exposure and recovery experiment, the cells were exposed to 3 or 10 μM MS-275 until they reached confluency (2-3 days), fed fresh media without drug for 24 h, and then dosed with 100 μM ZnSO_4_(Sigma-Aldrich) for 24 h and harvested for RNA isolation.

### RNA isolation and RT-PCR analysis

Total RNA was isolated from the cells according to the protocol supplied with TRI REAGENT (Molecular Research Center, Inc., Cincinnati, OH) as described previously by this laboratory [16]. Real time RT-PCR was used to measure the expression level of MT-3 mRNA levels utilizing a previously described MT-3 isoform-specific primer [16]. For analysis, 1 μg was subjected to complementary DNAsynthesis using the iScript cDNA synthesis kit (Bio-Rad Laboratories, Hercules, CA) in a total volume of 20 μl. Real-time PCR was performed utilizing the SYBR Green kit (Bio-Rad Laboratories) with 2 μl of cDNA, 0.2 μM primers in a total volume of 20 μl in an iCycler iQ real-time detection system (Bio-Rad Laboratories). Amplification was monitored by SYBR Green fluorescence and compared to that of a standard curve of the MT-3 isoform gene cloned into pcDNA3.1/hygro (+) and linearized with *Fsp *I. Cycling parameters consisted of denaturation at 95°C for 30 s and annealing at 65°C for 45 s which gave optimal amplification efficiency of each standard. The level of MT-3 expression was normalized to that of β-actin assessed by the same assay with the primer sequences being sense, CGACAACGGCTCCGGCATGT, and antisense, TGCCGTGCTCGATGGGGTACT, with the cycling parameters of annealing/extension at 62°C for 45 s and denaturation at 95°C for 15 s. Semiquantitative RT-PCR was also performed for MT- 3 expression using the GeneAmp RNA PCR Kit (Applied Biosystem, Foster city, CA) as described previously [17].

### ChIP (Chromatin Immunoprecipitation) assay

ChIP assays were carried out using the ChIP-IT™ Express kit (Active Motif, Carlsbad, CA). The protocols and reagents were supplied by the manufacturer. UROtsa parent and the transformed cell lines were seeded at 10^6 ^cells/75 cm^2 ^flask and 24 hrs later treated with 10 μM MS-275. Following incubation for 48 hrs, the cells were fixed with 1% formaldehyde for 10 min. Cross linking was stopped by the addition of glycine stop solution (0.125 M). The cells were scraped in 2 ml phosphate buffered saline containing 0.5 mM PMSF. The cells were pelleted and resuspended in ice cold lysis buffer and homogenized in an ice-cold dounce homogenizer. The released nuclei were pelleted and resuspended in a digestion buffer supplemented with PMSF and protease inhibitor cocktail. The chromatin was sheared using the enzymatic shearing cocktail at 37°C for 5 min to an average length of 200-1500 bp. Approximately 7 μg of sheared chromatin was used to coat the protein G-coated magnetic beads along with 3 μg of the antibody. The following antibodies were used in the immunoprecipitations: MTF-1 (Santa Cruz Biotechnology, Inc., Santa Cruz, CA), Histone H3 trimethyl Lys9, Histone H3 trimethyl Lys4, Histone H3 trimethyl Lys27, (Active Motif) and Anti-acetyl-Histone H4 (Millipore, Billerica, MA). The negative control IgG was purchased from Active Motif. The coating was performed overnight at 4°C following which the beads were washed and the immune complexes were eluted using the elution buffer and the cross linking was reversed using the reverse cross linking buffer. The immunoprecipitated DNA was analyzed by real time PCR using the iQ™ SYBR Green Supermix kit from Bio-Rad and semi quantitative PCR using the Gene Amp PCR core kit from Applied Biosystems. The primers for the MT-3 promoter were designed to span certain segments of the MT-3 promoter as depicted in Figure 4, and the sequences and annealing temperatures are indicated in Table 2. For quantitative PCR analysis, the quantity of the PCR template found in each specific precipitate was normalized to the amount of the corresponding DNA sequence found in the fragmented chromatin solution present before antibody-based precipitation (normalized to percentage of DNA input).

**Table 2:** Sequences and PCR conditions for primers

Promoter regions	Upper primer	Lower primer	Product size (bp)	Annealing temperature °C
**MRE A & B**	AAAGAGCGGGCGCGGTGC	GACGCGCGGCTTGGCTAGTGG	111	75

**MRE C**	GGCCCCGGCAGTGCACA	GCGCACGCACTGCATCTGTCG	55	75

**MRE E, F, G**	ATGGTACGTGCGCGCTTCC	CATCCGCGTGCACGACCCACT	124	70

**Region 1**	GAACAGATCTGGCGTCCTG	GCGCACGTACCATCTCCGA	111	63

**Region 2**	GTCGGGCTCATCGTGA	ATTCTCCAGGACGCCAGAT	77	61

### Urinary cytology and immunostaining for MT-3

The collection of urine and access to clinical data was reviewed and approved by both the IRB at the University of North Dakota and the IRB of Sanford Health. All participants signed an informed consent document. The procedures for the collection of urine and preparation for urinary cytology were identical to those procedures used for clinical diagnosis of urinary samples in the Sanford Health Urology Clinic and the Sanford Health Cytology Laboratory in Fargo, ND. The Sanford Health Laboratory is fully accredited by the College of American Pathologists (CAP) and meets all standards of the Clinical Laboratory Improvement Act (CLIA). Briefly, urine samples were accessioned with time and date stamp upon arrival in the laboratory. Color, clarity and amount were recorded for each sample. The sample was centrifuged for 5 min at 2,000 rpm (Eppendorf 5810R) and the specimen decanted, leaving cellular material and 2 - 5 ml of supernatant. An equal volume of PreservCyt was added and 2 to 5 ThinPrep® slides prepared from each sample. The slides were spray fixed immediately after preparation and allowed to dry completely. Prior to immunostaining, sections were immersed in preheated Target Retrieval Solution (Dako, Carpinteria, CA) and heated in a steamer for 20 minutes. The sections were allowed to cool to room temperature and immersed into Tris-buffered saline containing Tween 20 for 5 minutes. The immunostaining was performed on a Dako autostainer universal staining system. A primary anti-rabbit MT-3 antibody generated and characterized by this laboratory was used to localize MT-3 protein expression [2,18]. The primary antibody was localized using the Dakocytomation EnVision+ System-HRP for rabbit primary antibodies. Liquid diaminobenzidine was used for visualization (DakoCytomation liquid DAB substrate chromogen system). Slides were rinsed in distilled water, dehydrated in graded ethanol, cleared in xylene, and coverslipped. The presence and degree of MT-3 immunoreactivity was judged by two pathologists. Sections of human kidney served as a positive control for MT-3 staining.

### Statistics

Statistical analysis for the promoter studies consisted of ANOVA with Tukey *post-hoc *testing performed by GraphPad PRISM 4. All statistical significance is denoted at *p *< 0.05.

For the urine cytology experiments, statistical analysis was performed with the aid of PASW Statistics 18 (SPSS, Inc., Chicago). Pearson Chi-square was used to calculate the distribution of MT-3 positive or negative counts in each group, as well as to evaluate the correlations of frequency of MT-3 positive or negative between each group. Kaplan-Meier method was applied for survival analysis, Log-rank and Tarone-Ware tests were used to analyze for statistical significance. A value of *p *< 0.05 was considered statistically significant.

## Competing interests

The authors declare that they have no competing interests.

## Authors' contributions

SS: Cell Culture and MRE ChIP Analysis. SG: Histone Modifications. CT: Informed Consent, Patient Urine Samples, Clinic Data. XDZ: Interpretation of Urinary Cytologies. YZ: Statistics and Patient Database. AA: Measurement of MT-3 Expression. MAS: Interpretation of Urinary Cytologies. DAS: Wrote the paper, Designed Study, Data Interpretation, IRB monitoring. All the authors read and approved the final manuscript.
